# Effect of a Soft Robotic Sock Device on Lower Extremity Rehabilitation Following Stroke: A Preliminary Clinical Study With Focus on Deep Vein Thrombosis Prevention

**DOI:** 10.1109/JTEHM.2019.2894753

**Published:** 2019-03-22

**Authors:** Fan-Zhe Low, Jeong Hoon Lim, Jeevesh Kapur, Raye Chen-Hua Yeow

**Affiliations:** Department of Biomedical EngineeringNational University of Singapore 37580 Singapore119077; Department of MedicineNational University of Singapore 37580 Singapore119077; Singapore Institute for Neurotechnology, National University of Singapore 37580 Singapore119077; Advanced Robotics CenterNational University of Singapore 37580 Singapore119077

**Keywords:** Clinical trial, inpatient, physical therapy, rehabilitation, soft robotics, stroke

## Abstract

Background and objective: Immobility of the lower extremity due to medical conditions such as stroke can lead to medical complications such as deep vein thrombosis or ankle contracture, and thereafter prolonged recovery process of the patients. In this preliminary clinical study, we aimed to examine the effect of a novel soft robotic sock device, capable of providing assisted ankle exercise, in improving blood flow in the lower limb to prevent the complication of strokes such as deep vein thrombosis and joint contracture. Methods: Stroke patients were recruited (*n* = 17) to compare patients using the conventional pneumatic compression device with our robotic sock device on separate days. The primary outcome was to compare the venous flow profile of the superficial femoral vein in terms of the time average mean velocity and volumetric flow. The secondary outcome was to identify the ankle joint range of motion with the assistance of the device. Results: We noted improvements in the venous profile at the early phase of the device use, though its efficacy seemed to drop with time, as compared to the IPC device, where there was a significant improvement in the venous profile. The ankle joint dorsiflexion-plantarflexion range of motion assisted by the device was 11.5±6.3°. Conclusion and clinical impact: The current version of our sock device appears to be capable of improving venous blood flow in the early phase of device use and assisting with ankle joint exercise. The insights from this preliminary clinical study will serve as the basis for further improvement of the device and subsequent conduct of a longitudinal clinical trial. Funding: National Health Innovation Centre Singapore (NHIC) grant, R-172-000-391-511, MOE AcRF Tier 1 R-397-000-301-114.

## Graphical abstract

In this preliminary clinical study, we tested the effect of a novel soft robotic sock device, capable of providing assisted ankle exercise, in improving blood flow in the lower limb for the prevention of complications of stroke such as deep vein thrombosis and joint contracture. The results of the clinical trial show that the sock device improves venous blood flow in the early phase of device use and assists with ankle joint exercise.

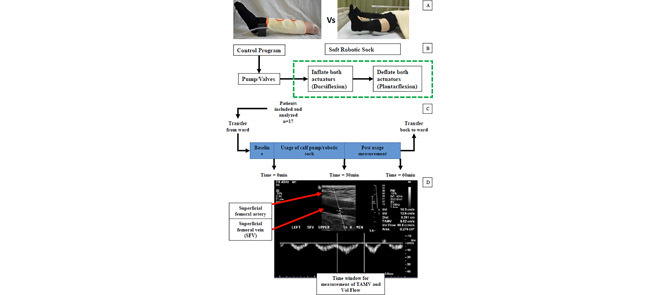

## I. Introduction

Stroke is one of the top medical conditions resulting in high mortality among patients in hospitals [1]–[3], where complications related to immobility such as deep vein thrombosis (DVT) [4] or ankle spasticity [5] can affect patients in their road to recovery. Patients may often take months or even years to fully recover their limb functions, where certain activities of daily living cannot be easily achieved prior to recovery. In the case of the affected lower limbs, immobility can affect common activities such as getting out of bed or commuting short distances [6].

Traditional physical therapy can be implemented to aid in motor recovery, where such therapy can be implemented through intended rehabilitation sessions with range of motion exercises conducted by physiotherapists on the joints [7] or through mechanical devices [8], [9] such as the continuous passive motion device intended to mobilize the paretic foot through hard robotic control. Although intended to be provided as early as possible for such stroke patients so as to prevent immobility complications [10], [11], such therapy sessions are difficult to be implemented effectively due to issues such as being manpower and resource intensive, coupled with the fact that such therapy require significant cooperation between patients and physicians [12]. Therefore, conventional physical therapy cannot provide effective benefits for stroke patients to recover.

In relation to the time of implementation of such physical therapy, there is also debate on the effectiveness of an early rehabilitation regime, such as within 3 days after admission for stroke [13]. In the same trial, it was shown that early and intensive rehabilitation was able to improve recovery functions in the lower limb in the case of patients with ischemic stroke, as well as to enhance early mobilization so as to prevent secondary infectious complications related to stroke [14]. In other trials where very early mobilization within 24 or 48 hours of admission was tested [15], [16], it was found that such procedure was efficient to promote motor recovery, especially with 2 to 3 times of therapy conducted per day. However, some of the stroke patients are often bedridden during the early phase of admission and coupled with the fact that these patients normally have other associated medical conditions [17], intensive rehabilitation will be hard to implement. This is especially true in the case of hemorrhagic stroke patients where patients may not be allowed to go for physical therapy in the early phase of subacute stroke recovery.

In earlier studies, we developed a soft robotic sock device [18], [19] where the device is able to provide bed-top rehabilitation for initially-admitted stroke patients so that they can continuously receive robot-assisted mobilization of the lower limbs. We hypothesize that with mobilization of the ankle joint with soft actuators to simulate natural ankle dorsiflexion and plantarflexion movement, we can promote clinical prevention of DVT with promoting blood circulation and prevention of ankle spasticity with mobilization of soft tissues around the ankle joint, where immobility of the lower limb is a major factor.

The goal of this preliminary clinical study was to evaluate the efficacy of the soft robotic sock device in providing robot-assisted ankle dorsiflexion-plantarflexion and improving venous flow during inpatient usage, when compared to conventional mechanical prophylaxis device (Intermittent Pneumatic Compression device) that solely aims at improving venous flow to prevent DVT [20]. The study collected ultrasound Doppler data to determine the primary outcome of venous flow activity in the lower limbs over 2 separate sessions when devices were worn on the lower limbs of stroke patients. The secondary outcome of assisted range of motion with the sock device is also presented.

## II. Methods and Procedures

### A. Study Design and Human Subjects Protocol

Patients admitted at a local hospital with a diagnosis of either ischemic or hemorrhage stroke, were eligible for this study. Age requirement for this study was fixed at 40 to 100 years old. Criteria for recruitment also includes patients with severe lower limb weakness based on the Medical Research Council (MRC) scale of less than 3 in the flexors and extensors of the knee and ankle, which was a measure of the motor strength as conducted by physicians. The comprehensive inclusion and exclusion criteria are presented in Table 1. This study was approved by the National Healthcare Group Domain Specific Review Board (DSRB, reference number 2014/00802) with data collected at the Department of Diagnostic Imaging at the local hospital. Prior to the study, informed consent were obtained from all patients.

**TABLE 1:** Inclusion and Exclusion Criteria for the Clinical Study

Inclusion Criteria (all to be met):	
•	Ischemic or hemorrhagic stroke patients
•	Signed informed consent prior to inclusion into the study
•	Aged range between 40 and 100
•	Severe lower limb weakness (MRC scale <3) in flexors and extensors of the knee and ankle
Exclusion Criteria:	
•	Medically unstable patients
•	Queried deep vein thrombosis cases
•	Limited range of motion in ankle and foot including equinus or club foot deformity
•	Musculoskeletal pathology in lower limbs
•	Serious injury to foot, ankle, knee and hip

**FIGURE 1.**
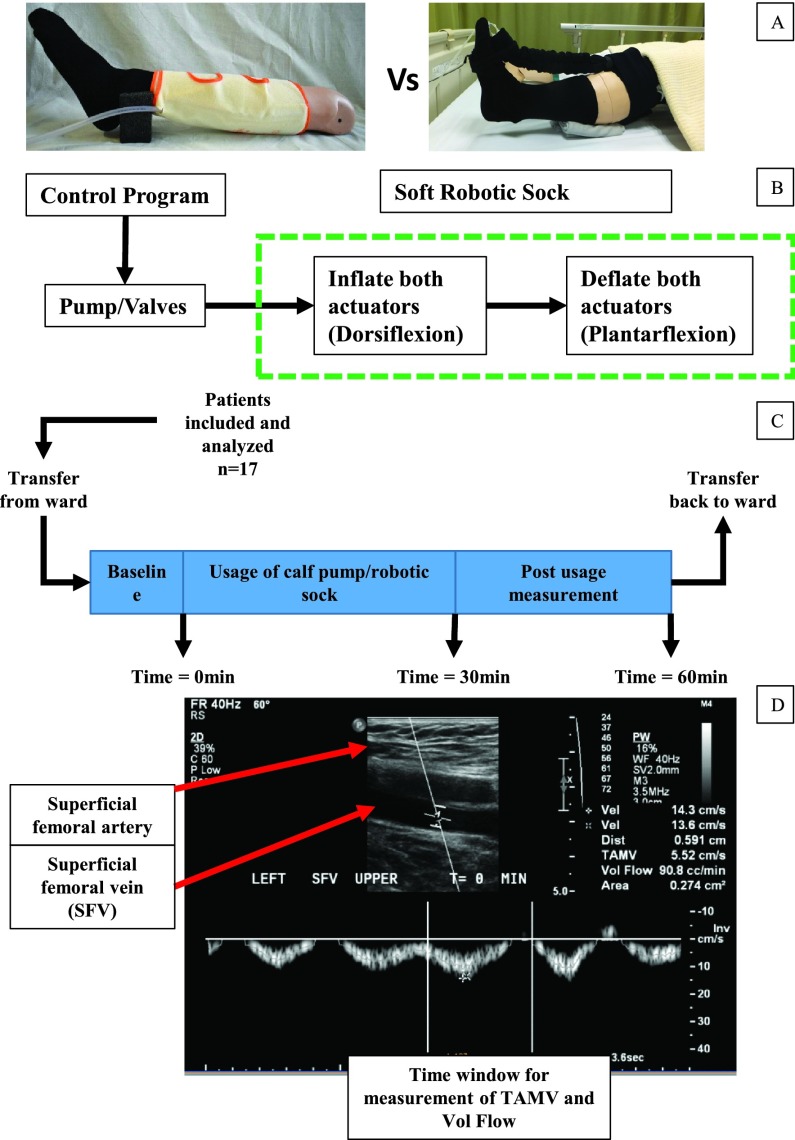
Schematic of the flow of the study. (A) Intermittent pneumatic compression device with the calf sleeve worn around the calf and attached to a pneumatic pump (Left) and Soft robotic sock device worn on the lower limbs with pneumatic actuators used for dorsiflexion-plantarflexion of the ankle (Right). Both devices were worn on the ipsilateral limb on alternate days. (B) Schematic of the robotic sock with pneumatic pump-valve control. (C) Sequence for each session of clinical trial attended by the patient with baseline measurement (0min), measurement during usage of either devices (10, 20, 30min) and post usage measurement (40, 50, 60min). Patients are transferred to and from the imaging room via trolley. (D) A set of ultrasound image identifying the superficial femoral artery and targeted superficial femoral vein (SFV) for venous flow measurement. The labelled time window is used to determine the time averaged mean velocity (TAMV) as well as volumetric flow of the SFV.

The study followed a cross-over design where patients were required to attend two sessions conducted on alternate days (Figure 1). On one session, the patients wore the conventional intermittent pneumatic compression (IPC) device (Flowtron, Arjohuntleigh, Sweden) on the affected leg, which compressed the calf for 12s at 40mmHg, with 1 cycle per minute. On the second session, the patients wore our soft robotic sock device [18], [19] which provided robot-assisted ankle dorsiflexion-plantarflexion by controlling the internal pneumatic pressure of the soft extension actuators, with the device running at 3 cycles per minute repetitively. Each cycle of ankle flexion consisted of 10s of dorsiflexion with deflation of soft actuators (tensile in actuators pulled ankle into dorsiflexion) and 10s of plantarflexion with inflation of soft actuators (relaxation and extension of actuators guide ankle to plantarflexion). For both sessions, the devices were worn for 30min, with data collected for baseline before trial (0min), during usage of device (10, 20, 30min) and post usage of device (40, 50, 60min).

The implementation of the soft robotic sock device requires a knee brace and sock worn on the lower limb. The knee brace worn around the whole knee prevent significant slippage during tensioning of actuators into dorsiflexion. Two pieces of the soft actuators placed within a denim fabric to prevent excessive radial expansion were attached to the foot (metatarsal region) and knee brace (distal of knee joint) at the two ends. The actuators pull the ankle into dorsiflexion when deflated due to tension and guide the ankle into plantarflexion when inflated and extended due to the resistive torque in the ankle. The duration of pneumatic actuation was controlled by a micro-controller (Arduino, Italy) coupled with a pneumatic pump-valve system (Parker, USA).

### B. Outcome Measures

The primary outcome measure was to determine the venous flow in the ipsilateral lower limb measured using ultrasound Doppler at the Department of Diagnostic Imaging (National University Hospital, Singapore), with triplicate data captured at an interval of 10min. Measurements were taken on the superficial femoral vein (SFV), 2cm away from the bifurcation with the deep femoral vein. With the usage of the IPC device, data was collected when the calf sleeve was pressured within the 12s cycle. On the other hand, for the soft robotic sock device, data was collected when the actuators were inflating and the ankle was plantarflexing. The blood flow data will be presented with the time averaged mean velocity (TAMV) and volumetric flow.

The secondary outcome measure was to determine the range of motion of the ankle provided by the soft robotic sock device, which the IPC was not able to do so. Videos were taken when the sock device was working on the lower limb and analysed using an open-sourced software (Tracker Video and Modelling Tool, Cabrillo College, US), where the software was used to track three distinct points on the lower limb to determine the robot-assisted ankle joint range of motion. The data was plotted for range of motion of the ankle against percentage of flexion cycle to reflect the motion over time with assistance from the soft robotic sock device. However, as the ankle maximum range of motion occurred at a different time point for each subject, peak to peak comparison for each subject was made to present the average peak values for the range of motion.

### C. Statistical Analysis

We used the paired one-tailed t-test to compare the ultrasound data between each unique time point (10-60min) with the baseline data (0min). All statistical analyses were performed using OriginPro Version 9.1 (OriginLab, USA). Values of P<0.05 were considered significant in our analysis.

## III. Results

### A. Study Participants and Baseline Characteristics

In total, 17 patients were screened and recruited (Table 2). The mean age of the participants was 57.8± 14.6 years, with 11 male (65%) and 6 female subjects (35%). The average time between admission and recruitment was 16± 10 days. Out of all the subjects, 3 had ischemic stroke (18%) while 14 had haemorrhagic stroke (82%), either with initial haemorrhagic diagnosis or haemorrhagic conversion from ischemic stroke. The initial assessment for motor function using the Medical Research Council (MRC) scale for muscle strength for the patients were 1.2± 1.3, based on the inclusion criteria of patients with MRC less than 3.

**TABLE 2:** Baseline Characteristics of the Patients

Characteristics	Clinical trial group, n=17
Sex, M/F	11/6
Age, mean (SD)	57.8 (14.6)
Time between admission and recruitment (days), mean (SD)	16 (10)
Stroke	
Ischemic/hemorrhagic	3/14
Affected side of limb, left/right	12/5
Medical Research Council (MRC) Scale for muscle strength, mean (SD)	1.2 (1.3)

### B. Primary Outcome

**FIGURE 2.**
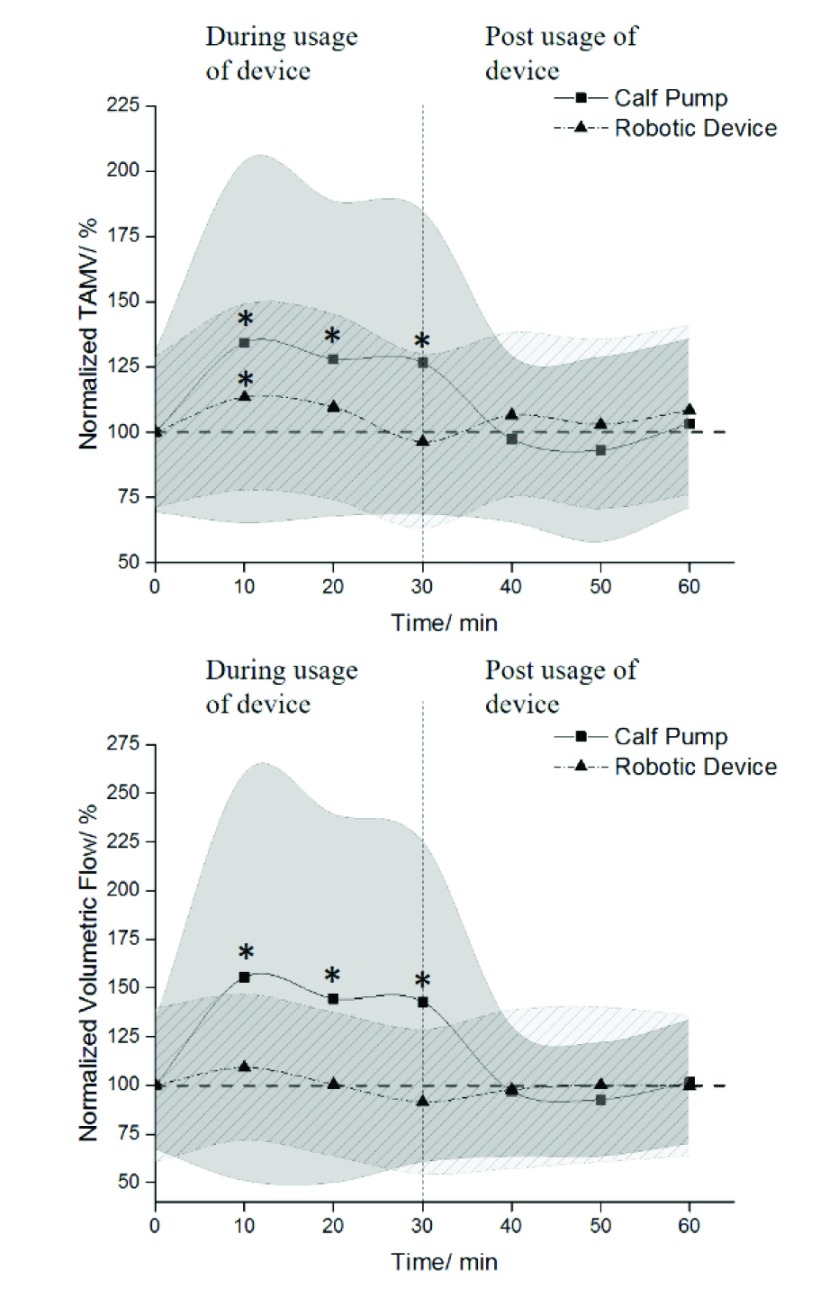
Graphs of TAMV and volumetric flow with values normalized to baseline value. * shows a significant difference when compared with baseline value, with values of P<0.05 considered significant in our analysis. The plotted data points represented the averages of the normalized values with the shaded region representing the standard deviation.

The TAMV showed significant increase in venous velocity (time = 10 min, 8.74± 4.50cm/s, P=0.011; time = 20min, 8.33± 3.93cm/s, P = 0.019; time = 30min, 8.24± 3.79cm/s, P = 0.006) during the use of IPC, when compared with baseline value (6.51± 2.01cm/s). The TAMV showed significant increase in venous velocity (6.36± 2.00cm/s, P = 0.048) during the soft robotic sock device at 10min, when compared with baseline value (5.61± 1.61cm/s). At 20min, the TAMV increased (6.15± 2.00cm/s, P = 0.107) and at 30min, the TAMV decreased (5.40± 1.88cm/s, P = 0.310) (Figure 2).

The volumetric flow showed significant increase in venous flow (time = 10min, 305.9± 205.9cm^3^/min, P = 0.008; time = 20min, 284.3± 186.6cm^3^/min, P = 0.010; time = 30min, 281.1± 162.4cm^3^/min, P = 0.004) during the use of IPC when compared with baseline value (196.8± 65.0cm^3^/min). Analysis of volumetric flow showed increase in venous flow at 10min (209.6± 71.8cm^3^/min, P=0.086) and at 20min (192.9± 71.0cm^3^/min, P=0.480) during the use of the soft robotic sock device, when compared with baseline value (192.1± 76.1cm^3^/min). At 30min, the volumetric flow decreased (175.5± 71.4cm^3^/min, P = 0.144) (Figure 2).

### C. Secondary Outcome

**FIGURE 3.**
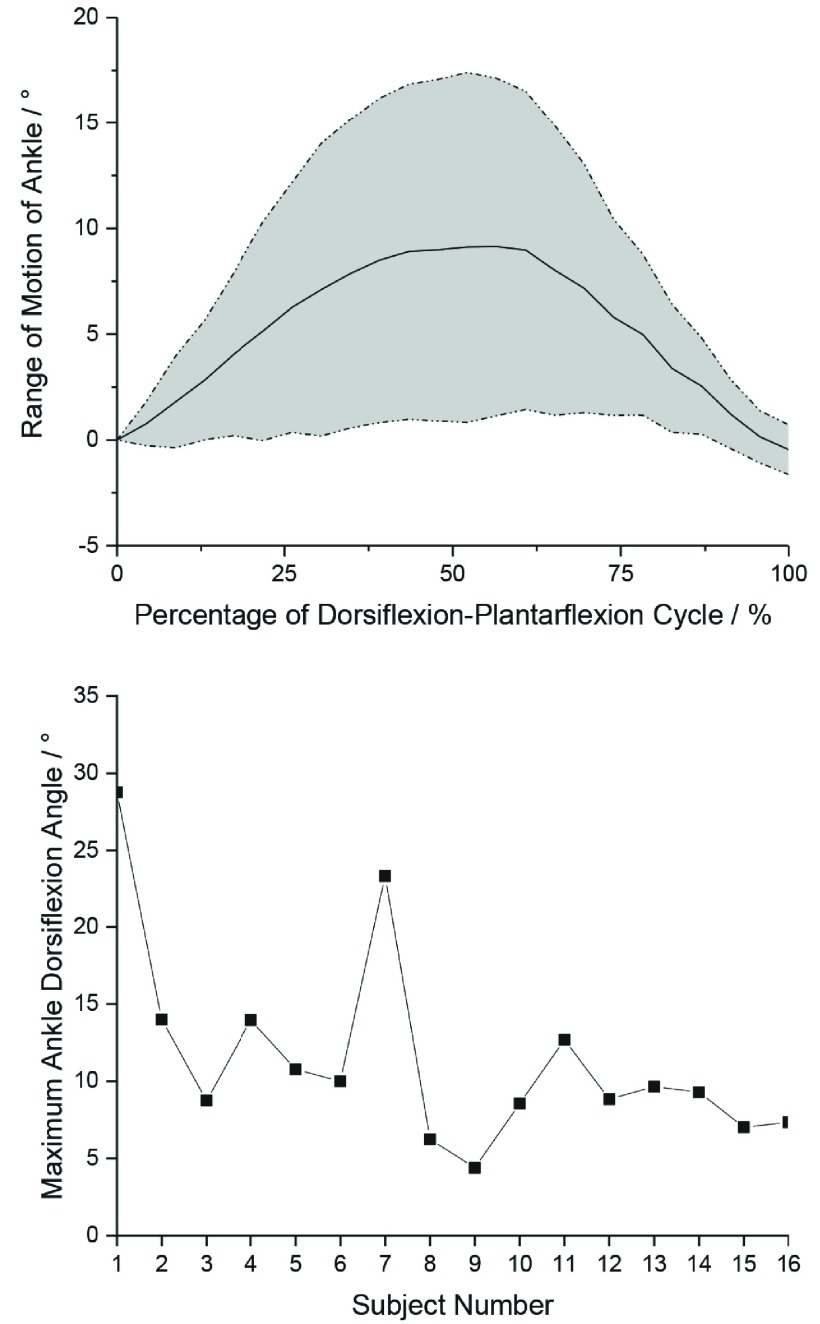
(Top) Average range of motion of the ankle across 16 patients. Values are presented versus percentage of dorsiflexion-plantarflexion cycle. The plotted data points represented the averages of the range of motion values with the shaded region representing the standard deviation. (Bottom) Maximum ankle dorsiflexion angle for the 16 subjects.

For the analysis of the ankle range of motion, only 16 subject data were analysed as one of the subject’s video was corrupted. The range of motion showed that the subjects achieved an average maximum ankle dorsiflexion angle of 11.5±6.3°, when comparing peak-to-peak angles across all subjects, with each subject reaching maximum robot-assisted ankle dorsiflexion at around 45-50% of each actuation cycle (after 9-10s of actuator deflation) (Figure 3). On the other hand, the IPC was not able to provide any form of assisted ankle dorsiflexion as it can only provide fixed compression of the calf at 40mmHg.

## IV. Discussion and Clinical Outcome Analysis

We conducted a pilot clinical trial on acute and subacute stroke patients (1-2 months post stroke, average time between admission and recruitment of 16± 10 days) by comparing a conventional mechanical prophylaxis device (IPC) with our soft robotic sock device in promoting venous blood flow, as well as providing assisted ankle dorsiflexion-plantarflexion. Our device showed early effectiveness in terms of its improved venous blood flow but lack sustained flow improvement over time.

Our use of Ultrasound Doppler emulates other studies that utilize ultrasonography to determine the effects of mechanical prophylaxis such as using devices or physical therapy on the lower limb [21]–[23]. In these studies, different veins were identified for ultrasonography measurement. In the case for DVT assessment while the patients were in the supine position using the rehabilitation devices, we were looking at the mapping of deep veins in the lower limbs. As we were not able to map the popliteal vein or any other vein distal to the popliteal vein without causing significant discomfort to the patients with tilting of the leg, we chose to conduct ultrasonography measurement on the superficial femoral vein, which was one of the veins that radiologists conduct ultrasound measurement to detect occurrence of DVT in conventional assessment.

For our secondary assessment, we used Tracker software for post analysis of joint ankle motion. However, for typical joint assessment, the gold standard is to conduct assessment in the gait laboratory with markers placed on the anatomical joints. In this case, we were not able to replicate the gait laboratory environment in the radiology room and therefore used a post analysis method for assessment of assisted ankle joint motion with our soft robotic sock device.

During the conduct of the preliminary clinical trial, there was no immediate feedback from the patients in terms of the functionality of the robotic device as this was a short-term usage study where patients were unable to feel their limbs. Moreover, the patients did not express discomfort from mobilization of the limbs during device control during this single session usage over 30 minutes.

For the IPC device, the parameters were set as advised by the manufacturer, similar to the parameters that patients received while using the device in the ward. For the robotic sock device, the parameters were provided taking into account the pressure required to fully inflate the actuators, as well as the pump specifications that were able to meet that pressure. In this case, the time taken for full inflation and deflation was 10s with the electronic setup used for this preliminary clinical trial. With no rest in between cycles, the electronic was then able to provide 3 cycles of dorsiflexion-plantarflexion per minute. In this case, for our soft robotic sock device, there could also be future improvement to the electronics setup to allow for faster assisted dorsiflexion and plantarflexion of the ankle.

One limitation of the soft robotic sock device is the implementation of fixed electronic parameters such as fixed duration of inflation-deflation of actuators for dorsiflexion and plantarflexion and fixed device dimensional parameters to all the patients involved in this study. In this case, fixing some of these parameters were ineffective in providing effective assisted actuation of the ankle, since some patients may experience stiffer ankle joint, which could be correlated to gender, age and even anthropometric parameters of the body.

Another limitation was the small sample size of stroke patients who participated in our clinical trial, which may not be representative of the whole population of stroke patients with large inter-subject variability [1], [24]. Therefore, the ratio for ischemic and hemorrhagic ratio presented in this study may not be typical of the stroke population as patients were recruited if they fulfill the inclusion and exclusion criteria. Factors such as type of stroke, neural injury or other clinical factors such as age and gender can affect the patient’s compliance to using our robot-assisted device for ankle exercise treatment. This can be identified in our baseline characteristics and supplementary figure of our patient group with high variability in medical diagnosis and identified clinical factors.

Even for the case of recruitment of the in-patient stroke patients, most of the patients had completed their acute care in hospital and were awaiting transfer to an inpatient rehabilitation facility. Therefore, most of the patients were not recruited within the phase of early rehabilitation [25] and factors such as spasticity might have developed and further decreased efficacy of the soft robotic sock device, since a spastic limb requires stronger force to conduct ankle flexions as well as extended duration of ankle mobilization, which was not available in this preliminary clinical trial which presented usage over a single session.

The novelty of this study is the implementation and evaluation of a soft robotic sock device for bed-top robot-assisted rehabilitation of stroke patients. The ultimate purpose of the soft robotic sock device was not to replace conventional mechanical prophylaxis devices or to replace physical therapy on the lower limbs, but rather to provide a supplementary rehabilitation approach with alternative mechanical prophylaxis that was able to provide early and intensive bed-top rehabilitation of the lower limb in the hospital wards. With the device, we can prevent immobility-related complications to the body, such as joint contractures and DVT simultaneously.

In an earlier paper, it was mentioned that early and intensive rehabilitation were independently associated with improved activities of daily living [13]. Several studies working on robotic devices showed repeated stretching and continuous passive exercise can increase blood flow, ankle range of motion and mobility function [26]–[30]. Therefore, the function of our soft robotic sock was to replicate the exercise, where in this case the device can provide extended duration of dorsiflexion and plantarflexion to the ankle joint. With this, the advantage over conventional therapy is that our device can be incorporated into bedside care where patients do not have to rely solely on physiotherapy sessions or conventional mechanical prophylaxes, where patients receive robot-assisted ankle actuation while resting on the ward bed during hospitalization and intensity can be controlled by varying electronic parameters for control.

With such a bed-top device that was able to provide assisted ankle actuation, future improvements to the device can also look into task-oriented approach of usage such as tele-rehabilitation or virtual reality [31], [32], where stimulating neural input during rehabilitation can provide a more effective outcome for stroke recovery. Such systems will require a more intricate sensorized system such as able to compute the absolute angle of the ankle to be recorded and displayed.

## V. Conclusion

The early version of our soft robotic prototype used in the ultrasonography pilot trial produced promising results for improving blood flow in the lower limbs of stroke patients and this will serve as the basis for improvement to the device to address any limitations identified. The improvements to the device can look into providing a more comprehensive actuation of the synovial ankle joint with possible three axes of motion. The initial step of implementing this ultrasonography clinical trial will serve as a basis to further conduct a longitudinal clinical trial during early-phase acute and subacute rehabilitation recovery with extended bed-top use, where comprehensive data pertaining to immobility such as DVT and ankle joint contracture can be collected.
